# Alcohol, betel-nut and cigarette consumption are negatively associated with health promoting behaviors in Taiwan: A cross-sectional study

**DOI:** 10.1186/1471-2458-13-257

**Published:** 2013-03-21

**Authors:** Su-Er Guo, Tung-Jung Huang, Jui-Chu Huang, Ming-Shyan Lin, Rei-Mei Hong, Chia-Hao Chang, Mei-Yen Chen

**Affiliations:** 1 College of Nursing, Chang Gung University of Science and Technology (CGUST); Director of the Chronic Diseases and Health Promotion Research Center, CGUST, Chang Gung, Taiwan; 2 Chang Gung Memorial Hospital, Yunlin, Taiwan; 3 Division of Endocrinology and Metabolism, Department of Internal Medicine, Chang Gung Memorial Hospital, Yunlin, Taiwan; 4 Division of Cardiology, Department of Internal Medicine, Chang Gung Memorial Hospital, Yunlin, Taiwan; 5 College of Nursing & the Chronic Diseases and Health Promotion Research Center, Chang Gung University of Science and Technology, No. 2, Chia-pu Rd. West Sec, Putz City, Chiayi County 61363, R.O.C. Taiwan

**Keywords:** Alcohol, Betel-nut, Cigarette, Nursing-led community health, Health promotion, Oral cancer

## Abstract

**Background:**

Oral cancer is the 2^nd^ most common cause of death due to cancer in the south-western coastal region of Taiwan; the standardized mortality of oral cancer is higher than elsewhere in the world. According to the evidence, alcohol, betel-nut and cigarette (ABC) consumption cause oral, nasopharyngeal and related cancers. This study describes the relationships between ABC consumers and health promoting behaviors among community adults living around an area with a high prevalence of oral cancer.

**Methods:**

A population-based, cross-sectional study design was conducted in oral cancer epidemic areas in south-western coastal Taiwan in 2010, 6,203 community residents over 20 years of age participated. Demographic data, ABC habits, and health-promoting behaviors were explored. A logistic regression analyses were used to identify factors associated with ABC consumers.

**Results:**

A high percentage of participants consumed alcohol, betel-nut and cigarettes. Betel-nut and cigarette consumers took low levels of exercise, adopted a poor diet, and had poor oral hygiene. After adjusting for potential confounders, the logistic regression model indicated that middle aged males of poor education and low economic status, who did not exercise regularly and had poor oral hygiene, were more likely to chew betel quid and smoke cigarettes.

**Conclusions:**

It has identified that BC consumers are negatively associated with health promoting behaviors. Further research is required to understand the reasons why the subjects consume ABC, and explore ways to prevent initiation and enhance cessation of ABC habits in this population.

## Background

### Nurse-led health promotion program to prevent chronic disease and related cancers

Each year the International Council of Nurses (ICN) provides a theme or mission on International Nurse’s Day for global nursing reflection and direction. During the last 5 years (2008–2012) themes have concerned serving communities, with nurses leading primary health care, chronic care and closing the gap from evidence to action [1]. These missions motivated community nurses in Taiwan to actively participate in providing health promotion programs, cooperating with public health nurses, school health nurses and faculty members from the schools of nursing to take a leading role concerning community health development. The World Health Organization (WHO) has said that “chronic diseases, such as heart disease, stroke, cancer, chronic respiratory diseases and diabetes, correspond to 63% of all deaths in the world” [2]. Alcohol and cigarettes contribute to these chronic diseases [3], and ABC consumption causes oral cancer [4]. In this study, the authors found that “the incidence of oral cancer is 123-fold higher in ABC consumption, compared to non-ABC consumption” [4]. Twenty years ago, 10% Taiwanese over 15 years old were ABC users [5]. In particular, betel nut trees became a significant agricultural product in Taiwan [6]. Therefore, reducing ABC consumption is increasingly important in promoting health.

According to ICD-10, oral cancer in the south-western coastal region of Yunlin County is ranked as the 2^nd^ most common cause of death from cancer in males, and its standardized mortality rate (34/100,000) is higher than that of the Taiwanese nation (14.9/100,000) and throughout the rest of the world [7]. Some of the mechanisms and specific risk factors associated with developing cancer remain unknown. However, the majority of factors associated with oral cavity-related cancers, including the oral sub-mucosa, tongue and nasopharynx, are related to alcohol, betel quid (areca nut) chewing and cigarette smoking [8-11]. Of these 3 habits, chewing betel quid, widespread in Southern Asian populations, is the major factor that causes oral cancer [10,12]. This habit is common in Taiwanese adolescents and adults with a poor educational level, and in minority populations in rural areas [12].

According to the mission of ICN, nurses are expected to serve communities and lead community health facilities. Therefore, this research team conducted a longitudinal study, based on community health assessment through a health promotion program, with the aim of reducing ABC consumption.

### Issues of ABC

Over-consumption of alcohol harms health and social relationships, and, according to [13], excess alcohol consumption results in 2.5 million deaths every year. It can result in cardiovascular diseases, liver cirrhosis, hypertension, diabetes, dyslipidemia, abdominal obesity, metabolic syndrome, various cancers and several psychosocial related issues [14]. Numerous studies show that alcohol consumption is strongly associated with morbidity, mortality, and social problems in developing and developed countries [1,14,15].

Chewing betel quid is significantly associated with adverse health effects, including oral and pharyngeal cancer, sub-mucosal fibrosis, gum disease, obesity, metabolic syndrome, hypertension and cardiovascular mortality [8,12,13]. Betel quid chewing is a common habit in Southern Asian populations, e.g. Malaysia, India, Pakistan, Sri Lanka, Myanmar, Thailand and Taiwan [8], and its contribution to the development of oral sub-mucosal fibrosis and oral cancer confirmed [9,16]. Retrospective studies indicated that many betel quid users combine this with alcohol consumption and cigarette smoking [7]. Cigarette smoking is responsible for several adverse cardiovascular and respiratory outcomes [17,18]. Combined with betel quid chewing it also has a significant contribution (86.5%) to the risk of oral leukoplakia and oral sub-mucosal fibrosis [16].

Despite numerous studies reporting that the mechanism of ABC habits are associated with oral cancer, those that focus on combining ABC habits with adopting health promoting behavior in areas with a high prevalence of oral cancer are scarce. The purpose of this article, therefore, has been to outline the first phase of a longitudinal study and decide whether ABC is associated with poor health promotion.

## Methods

### Design, sample and setting

This study is the first part of the longitudinal study of health promotion for community health development lead by nursing faculty members (HPCHN) around areas in south-western coastal Yunlin County, Taiwan, where oral cancer is endemic. Using a descriptive cross-sectional design, this community-based survey was implemented every Monday to Saturday from October 2010 to September, 2011. Of 47,798 residents living in 27 villages, the response rate was 14.5% (6,911). The participants were selected by convenience sampling at the villages in the south-western coastal areas. The inclusion criteria were adults: (1) aged 20 years and over, (2) fully independent in managing their daily lives, (3) able to complete the questionnaires in Mandarin or Taiwanese dialects either by self-administration or at interview, without serious mental problems, (4) able to walk to a community center or the corporate private hospital, and (5) who agreed to sign an informed consent before being enrolled in the study. A total of 6,203 community residents participated.

### Procedure and ethical considerations

This study was conducted with a corporate private hospital (135 beds) through the community health screening program, and approved by the institutional review board ethical committee (Chang-Gung Memorial Hospital Ethics Committee No 99-2501B). The interviewing procedures and privacy protection were explained to the participants by the research assistants. There were 2 stages in the set-up: (1) A systematic literature reviews needed to draw up the first version of the questionnaire. ABC were used as the major keywords to search PubMed, Ovid and the Chinese database from 1980 to 2011. (2) Face and content validity was judged to be good (CVI = 0.90–0.92) by a panel of 5 experts - public health and health education faculty members, metabolic physicians, and nursing faculty members teaching health promotion. Some items within the instruments were revised according to the experts’ suggestions.

All research assistants were trained for 4 hours by the investigators. Research assistants were senior nursing students in a Post-RN Bachelor of Nursing Degree Program who held registered nursing license and received 2 consequent training programs, each of 2 hours duration. In session 1, we focused on understanding research background and practicing interview skills. In session 2, 20 research assistants were grouped into 10 pairs to pre-test and be familiar with all items of the questionnaire. Finally, research assistants were divided 2 groups to interview each elder and a 90% correct rate of inter-rater reliability was confirmed among the 10 pairs. The questionnaire consisted of 2 sections and the characteristics of the participant, as described below.

### Instruments

*ABC* habits were measured using 5 questions: (A) ‘do you drink alcohol?’ Participants were classified as ‘less consumption’ if they had never drank alcohol or had not drank for one year, or ‘regular consumption’ if they were currently drinking’; (B) ‘do you chew betel-nut?’ Participants were classified as ‘less consumption’ if they had never chewed betel-nut or no longer chewed’, or ‘regular consumption’ if they were currently chewing one quid or more per day’ [4]; (C) ‘do you smoke cigarettes?’ Participants were classified as ‘less consumption’ if they rarely or never smoked or ‘regular consumption’ if they were a current smoker one cigarette or more per day’ [4]; (D) ‘when did you begin to consume alcohol, betel nut chewing or smoke cigarettes?’ if participants answered that they no longer partook of these 3 habits, they were asked question (E). ‘How long is it since you quit drinking alcohol, betel nut chewing or smoking cigarettes?’

*Health*-*promoting behavior* was measured using 5 items including physical activity, requiring participants to answer the questions: (A) ‘do you take regular exercise?’ Participants were classified as ‘not often’ if their answer was never or sometimes, or ‘often’ if they usually exercised for >30 min per day, 3 times per week, or 150 minutes per week. (B) Vegetable and fruit habits: ‘do you have 3 portions of vegetables and 2 portions of fruit every day? The answer was classified as ‘not often’ if they answered never or sometimes, and as ‘often’ if they usually had at least 3 portions or one and half bowl-sized portions of vegetables, and at least 2 portions or one bowl-size of fruit >5 days per week. (C) Regular dental check-up behavior: ‘do you generally go to the dental clinic for a check up every half-year?’ the answer was classified as ‘not regular’ if the answer was never or more than one year before visiting a dentist, or ‘regular’ if they answered that they attended regularly or at least within half a year’. (D) Teeth brushing behavior: ‘do you think that you should brush your teeth after meals?’ the answer was classified as ‘incorrect’ if they did not brush their teeth after meals and use dental floss at least once per day, or ‘correct’ if they brushed their teeth after meals and used dental floss at least once per day’. (E) Frequency of brushing teeth: ‘how many times do you brush your teeth per day?’

*Participants*’ *characteristics* were obtained through structured questions relating to age (year of birth), gender, marital status, economic status (3 levels were categorized as good, fair/not bad, poor/bad), and educational attainment (receiving educational year or graduated school level).

### Statistical analysis

Analysis was conducted using SPSS 17 statistical software (SPSS INC., Chicago, IL). Coded data were subjected to range and manual checks for accuracy. To compare personal factors or health promoting behavior in various ABC consumption groups, the Chi-square statistic for testing equality of proportions or rates was used. Final models using binary logistic regression for analyzing betel-nut and cigarette consumption were chosen, based on likely and relevant confounders after univariate analysis (candidate correlates were excluded from the final analyses if p > 0.05 in the univariate analysis with outcome variable). Statistical significance was set at *p* < 0.05.

## Results

### Demographics and health related behavior

According to the statistics on household registration, the total of adult residents living in the 27 villages was 47,798 [19]. As several worked out of these townships, the baseline questionnaire was sent to 6,911 residents; 708 (10.2%) were not recruited to this study due to being too young, and others were rejected as they were unavailable for interview. Of the remaining 6,203 participants, 83.4% had lived in the community for >20 years and 57% were female (3,501). The mean age was 49.4 (SD = 16.4, range 20–95) years, with 78% (4,819) being in the range from 20 to 64 years. More than half (3552, 58%) were not educated past middle school. The majority (78%) was married, 39% had no occupation, and 25.1% worked in farming or fishing. Twenty-one percent (1,268) rated their economic status as poor and difficult, while 78% considered it average.

More than one-third of the participants (2,153; 35%) reported that they regularly consumed alcohol, 17% (1,017) chewed betel-nut and 26% (1,576) smoked cigarettes. Furthermore, 14% (872) reported regularly consuming betel-nut and cigarettes, and 9% (522) regularly consumed ABC. The beginning mean (median, range) age of using ABC was 23 (20, 12–71), 24 (21, 13–65), and 21 years of age (21, 10–71), respectively.

### ABC consumption is associated with personal factors and health promoting behavior

There were few differences between the personal factors and health promoting behavior in the alcohol, betel-nut chewing and cigarette smoking groups. Table 1 shows that subjects at study entry who consumed alcohol regularly were predominantly male (p < 0.001), with a high school or college education (p < 0.001), below 64 years of age (p < 0.001), with good or fair economic status (p < 0.001), often adopting physical activity (p < 0.01), not consuming vegetable and fruit (p < 0.001), not attending regular dental check-ups (p < 0.001) and brushed their teeth infrequently (t = 5.4, 95% CI = 0.08 ~ 0.17, p < 0.001). The characteristics and factors associated with betel-nut chewing or cigarette smoking were comparable (Table 1).

**Table 1:** **Demographic characteristics**, **health promoting behaviors and alcohol**, **betel**-**nut and cigarette consumption**

**Variables**	**Alcohol**	**Betel-nut**	**Cigarette**
	**Consumption N (%)**		
	**Less**^**1**^**/Regular**^**2**^	**Less**^**1**^**/Regular**^**2**^	**Less**^**1**^**/Regular**^**2**^
Mean, median, range	23, 20, 12 ~ 71	24, 21, 13 ~ 65	21, 20, 10 ~ 71
Gender	*χ*^2^ =906.7***	*χ*^2^ =1077.1***	*χ*^2^ =1636.4***
Female	2880 (83.7) 560 (16.3)	3394 (97.8) 75 (2.2)	3283 (94.8) 180 (5.2)
Male	1160 (46.8) 1319 (53.2)	1780 (67.3) 866 (32.7)	1332 (49.8) 1341 (50.2)
Educational level	*χ*^2^ =267.6***	*χ*^2^ =147.7***	*χ*^2^ =131.8***
<=middle school	2542 (76.8) 770 (23.2)	2849 (82.5) 606 (17.5)	2652 (76.7) 804 (23.3)
=high school	658 (54.2) 557 (45.8)	1002 (79.4) 260 (20.6)	810 (63.7) 462 (36.3)
> = college	807 (59.6) 546 (40.4)	1288 (94.8) 70 (5.2)	1124 (82.3) 242 (17.7)
Age (years)	*χ*^2^ =257.5***	*χ*^2^ =29.1***	*χ*^2^ =57.0***
20 ~ 39	1229 (59.1) 852 (40.9)	1826 (86.6) 283 (13.4)	1541 (72.5) 585(27.5)
40 ~ 64	1683 (66.9) 832 (33.1)	2167 (81.7) 484 (18.3)	1945 (73.4) 705 (26.6)
65~	1138 (85.1) 200 (14.9)	1193 (87.0) 178 (13.3)	1141 (82.9) 235 (17.1)
Economic status^3^	*χ*^2^ =20.7***	*χ*^2^ =36.9***	*χ*^2^ =16.1***
Good	80 (70.8) 33 (29.2)	103 (88.8) 13 (11.2)	93 (80.2) 23 (19.8)
Fair/not bad	3091 (66.8) 1537 (33.2)	4092 (85.9) 673 (14.1)	3646 (76.2) 1137 (23.8)
Poor/bad	795 (73.8) 282 (26,2)	892 (78.8) 240 (21.2)	802 (70.8) 330 (29.2)
Physical activity^4^	*χ*^2^ =8.3**	*χ*^2^ =15.5***	*χ*^2^ =14.1***
Not often	2138 (69.9) 919 (30.1)	2630 (82.8) 545 (17.2)	2332 (73.2) 853 (26.8)
Often	1912 (66.5) 965 (33.5)	2556 (86.6) 400 (13.5)	2295 (77.4) 672 (22.6)
Vegetable and fruit	*χ*^2^ =34.1***	*χ*^2^ =25.5***	*χ*^2^ =50.6***
Not often	3506 (67.2) 1709 (32.8)	4536 (84.0) 863 (16.0)	4019 (74.2) 1400 (25.8)
Often	447 (79.3) 117 (20.7)	528 (92.0) 46 (8.0)	502 (87.6) 71 (12.4)
Dental check-up	*χ*^2^ =27.2***	*χ*^2^ =51.6***	*χ*^2^ =23.7***
Not regular	2158 (71.4) 863 (28.6)	2583 (81.8) 574 (18.2)	2309 (73.0) 852 (27.0)
Regular^5^	1584 (64.8) 859 (35.2)	2203 (88.7) 280 (11.3)	1963 (78.6) 533 (21.4)
Tooth brush	*χ*^2^ =0.4	*χ*^2^ =38.4***	*χ*^2^ =19.3***
Incorrect	2967 (68.0) 1395 (32.0)	3754 (82.9) 775 (17.1)	3356 (73.8) 1193 (26.2)
Correct	1083 (68.9) 489 (31.1)	1432 (89.4) 170 (10.6)	1271 (79.3) 332 (20.7)
Tooth brush^6^	t value = 5.4***	t value = 7.0***	t value = 8.2***
(mean, sd)	1.82 (0.7) 1.69 (0.6)	1.80 (0.7) 1.60 (0.7)	1.82 (0.7) 1.62 (0.6)
95% CI	.08 ~ .17	.15 ~ .27	.16 ~ .26

^1^ Less consumption: The amount of ABC consumption was never or less or quit more than one year.

^2^Regular consumption: The amount of ABC consumption was more than recommended by medical experts.

^3^ Self-perceived economic status.

^4^ Often: regular physical activity at least 30 minutes per day or 150 minutes a week.

^5^ Regular: dental check up/per half year, which coverage by National Health Insurance.

^6^ Mean frequency of tooth brush per day and 95% confidence interval.

* p < .05 ** p < .01 *** p < .001.

Table 2 shows that betel-nut and cigarette consumption was associated with males (*χ*^2^ =1761.2, p < 0.001) that had received less education (*χ*^2^ =169.9, p < 0.001), aged between 40–64 (*χ*^2^ = 44.5, p < 0.001), had poor economic status (*χ*^2^ = 34.4, p < 0.001), did not often adopt physical activity (*χ*^2^ = 20.1, p < 0.001), did not often consume vegetable and fruit (*χ*^2^ = 56.3, p < 0.001), did not regularly have dental check-ups (*χ*^2^ = 44.4, p < 0.001) and brushed their teeth infrequently (*χ*^2^ = 32.9, p < 0.001). The mean frequency of tooth brushing per day in the betel-nut and cigarette group was significant less than the other 2 groups (F = 38.6, p < 0.001, Table 2).

**Table 2:** **Betel nut and**/**or cigarette consumption associated with personnel factors and health promoting behaviors**

**Variables**	**Number of betel-nut and/or cigarette consumption**^**1**^			***χ***^**2**^
	**0**	**1**	**2**	
		**B**^**3 **^**user: 17% C**^**4 **^**user: 26%**	**BC user: 14%**	
Gender				1761.2***
Female	3228 (93.4)	206 (6.0)	23 (0.7)	
Male	1214 (46.1)	670 (25.5)	748 (28.4)	
Educational level				169.9***
<=middle school	2517 (73.2)	448 (13.0)	472 (13.7)	
=high school	784 (62.4)	238 (18.9)	235 (18.7)	
> = college	1113 (82.1)	182 (13.4)	60 (4.4)	
Age (years)				44.5***
20 ~ 39	1513 (72.2)	327 (15.6)	257 (12.3)	
40 ~ 64	1854 (70.3)	391 (14.8)	393 (14.9)	
65~	1086 (79.3)	160 (11.7)	124 (9.1)	
Economic status				34.4***
Good	89 (76.7)	18 (15.5)	9 (7.8)	
Not bad	3522 (74.2)	674 (14.2)	551 (11.6)	
Difficult/very bad	759 (67.5)	168 (14.9)	198 (17.6)	
Physical activity^2^				20.1***
Not often	2242 (71.0)	458 (14.5)	457 (14.5)	
Often	2211 (75.0)	420 (14.2)	317 (10.8)	
Vegetable and fruit				56.3***
Not often	3858 (71.8)	813 (15.1)	706 (13.1)	
Often	494 (86.4)	40 (7.0)	38 (6.6)	
Dental check-up				44.4***
Not regular	2199 (70.0)	479 (15.2)	463 (14.7)	
Regular	1911 (77.1)	334 (13.5)	233 (9.4)	
Tooth brush-				32.9***
Incorrect	3209 (71.2)	670 (14.9)	630 (14.0)	
Correct	1244 (77.9)	208 (13.0)	144 (9.0)	
Frequency of tooth brush				F = 38.6***
(mean, SD)	1.83 (0.7)	1.64 (0.6)	1.58 (0.6)	Scheffe
95% CI	1.80 ~ 1.85	1.58 ~ 1.70	1.53 ~ 1.64	0 > 1 > 2

^1^ 0 = none habit of betel-nut or cigarette consumption, 1 = one habit of betel-nut or cigarette, 2 = both habits of betel-nut and cigarette.

^2^ Often: regular physical activity at least 30 minutes per day or 150 minutes a week.

^3^B = betel-nut.

^4^C = cigarette.

* p < .05 ** p < .01 *** p < .001.

### Correlation of betel nut and cigarette consumption

The logistic regression model (Table 3) indicated that males were 9.8 times more likely have indulge in BC consumption than females (OR = 9.77, p < 0.001). Participants with middle and high school education were 1.65 (p < 0.001) and 2.5 (p < 0.001) times more susceptible to betel-nut and cigarette consumption regularly than those with a college education, respectively. Those aged 40–64 were 1.85 times (p < 0.01) more susceptible to regular betel-nut and cigarette consumption than those aged over 65, although there was a relationship between vegetable and fruit intake and BC consumption in univariate analysis (Table 2). However, after controlling other confounders, vegetable and fruit intake was not an important correlate in the logistic regression model. Subjects that were less physically active, did not attend regular dental check-ups, and incorrectly brushed their teeth were 1.25, 1.29 and 1.42 times more susceptible to BC habits than others, respectively.

**Table 3:** **The logistic regression of betel**-**nut and cigarette consumption and associated factors**

**Variables**	**β**	**Odds ratio**	**95% CI**	***P***
Gender				
Male	2.28	9.77	8.08 ~ 11.82	<.001
Female*				
Educational level				
<=middle school	.50	1.65	1.25 ~ 2.18	<.001
=high school	.91	2.49	1.93 ~ 3.22	<.001
> = college*				
Age (years)				
20 ~ 39	.85	2.34	1.72 ~ 3.19	<.001
40 ~ 64	.61	1.85	1.45 ~ 2.34	<.001
65 ~ *				
Economic status				
Poor/bad	.56	1.75	0.93 ~ 3.29	.08
Fair/not bad	.26	1.30	0.72 ~ 2.34	.39
Good *				
Physical activity				
Not often	.22	1.25	1.04 ~ 1.49	.013
Often*				
Dental check-up				
Not regular	.31	1.29	1.02 ~ 1.63	.001
Regular*				
Tooth brush				
Incorrect	.35	1.42	1.12 ~ 1.80	.004
Correct*				

*Note*: * as reference.

## Discussion

The findings indicate a high prevalence of ABC consumption among adult community residents around areas with a high incidence of oral cancer. Regular users of betel quit and cigarettes are associated with low social economic status and adopting a less health promoting lifestyle. ABC consumption was also significantly associated with males.

### High prevalence of ABC consumption among adult residents around high oral cancer areas

This study did not use random sampling, but a significantly higher percentage of ABC consumption, compared with national data, was identified. The prevalence of ABC consumption was identified in 35%, 17%, and 26% of participants, respectively. In addition, when considering the gender difference, males that regularly consumed ABC were 53.2%, 32.7% and 50.2%, respectively. These numbers were higher than the nation-wide percentages among both genders. In males aged over 18 years, the prevalence of current ABC behavior was 18.8, 13.0 and 35.0%, respectively [10]. These differences may be due to location and professions. Most people who live in south-western coastal Yunlin County are farmers and fishermen. Based on the finding of Lee et al. [20], the highest prevalence of betel quid chewing and cigarette smoking in Taiwan is in agriculture or fisheries. This study also found that 14% regularly consumed both betel quid and cigarettes, and 9% consumed A, B and C regularly. This finding is similar to Ko et al. [5], which shows that, despite the Taiwan government conducting several strategies during the last two decades, problems persist.

To compare these habits with other countries, Ghani et al. [8] reported that in Malaysian adults 8.2% were betel quid chewers, a habit that was more prevalent among females. Females >40 years old with Indian ethnicity and a history of smoking were likely to develop a quid chewing habit. Despite betel nut and cigarette consumption, the prevalence of alcohol consumption is similar to Germany and South Korea. Donath et al. [21] found higher alcohol consumption in rural than urban areas in Germany, and Chung et al. [22] found that of the males, 90% are likely to drink excess alcohol in South Korea.

The age of starting to use ABC was very young - 12 years old for drinking alcohol, 13 for betel nut chewing and 10 for smoking cigarettes. Moreover, many betel nut users become cigarette smokers and alcohol drinkers. In the last 10 years, the Taiwan government incorporated several strategies to reduce the prevalence of ABC consumption, including legislation to increase the price or tax [7]. However, it is unknown why western coastal adults living around areas with high incidence of oral cancer partake of these 3 unhealthy habits. Do they know the etiology and mechanism(s) of oral cancer associated with ABC? Do the health and education policies penetrate into rural areas and the socially economic disadvantaged community residents? This may suggest that to understand more fully the complexity of consumption of ABC; investigators need to develop and use sensitive measures that can capture the multidimensional aspects of ABC behavior in these areas.

Participants who chewed betel-nut and consumed cigarettes tended to be male, less well educated, middle-aged, with poor economic status and an unhealthy life style (Table 2). These findings are similar to the results of Shieh et al. [23] who described the prevalence of chewing betel-nut and cigarette smoking in the general population 18 or more years of age in Chiayi city, a south-western coastal city of Taiwan. Based on this finding, the researchers will develop culturally and linguistically competent health education materials for this group in the future.

This study shows an inconsistent finding in the logistic regression model that alcohol users tend to have a better education, higher economic status, adopt more physical activity and receive regular dental check-ups. This phenomenon could be due to the questionnaire not including measuring and quantifying alcohol type, such as the brands of wine they drank, although beer is popular nation-wide with the percentage of alcohol being below 5%. Small volumes of alcohol may have positive effects on health [15]. The consequence could explain the false higher figure of alcohol consumption in this study. Therefore, we suggest a further study to consider the issue of health literacy and adequate ABC information, culture-oriented educational material or adapted language-sensitive pamphlets, and that it should take the alcohol type into account for middle aged community residents in regions with a high prevalence of oral cancer.

### More specific health promotion programs are necessary initiated in the western coastal rural areas

Comparing the data with nationwide statistics, participants exercised (56.5 vs. 48%) and used dental floss (55.4 vs. 39%) less often than the general population [7]. Betel nut and cigarette users also used dental floss significantly less (9.0%) and took less exercise (14.5%) than those without these 2 habits (Table 2). Oral health and exercise are recognized as important health-related behavior conducive to good mental and physical well-being [7,13]. Experts recommend brushing teeth often or at least twice a day, flossing teeth daily and regularly attending dental check-ups every 6 months [13]. Professional oral health resource and access to dental services are limited in rural areas. Therefore, community nurses should conduct health promoting programs related to the low cost, but effectiveness, of oral hygiene and physical activity through community-based health development, specifically for the social economic minority and males in rural areas.

In March 1995, Taiwan’s national health insurance (NHI) program was set up with the goal of providing high quality, affordable healthcare to all (NHI covers 99% of the population) [24]. However, in the last 5 years, financial difficulties and conflicts between government and the public led to the NHI program becoming unstable. Enhancing health promoting programs might reduce the health-related incidence of chronic disease. Furthermore, national statistics showed that life expectancy in Yunlin County is 3 years less in males compared with Taiwan population (73.4 vs. 76.1 years) [24]. It is common for men to die at a younger age than women worldwide [1], but the average is 6 years in Taiwan (76.1 vs. 82.6 years) [24].

Several studies have indicated that it is not only the male gender that is associated with these 3 kinds of behavior [22,25], but that socioeconomic inequalities are important [10,26,27], with poor education, low economic status and living in rural areas being associated with ABC use [21,26]. Our findings indicate that 4.5% (122) of alcohol, 13.4% (360) of betel nut, and 13.3% (357) of cigarette users reported abstinence for >1 year. Therefore, it is possible to initiate health promoting programs to reduce the prevalence of ABC behaviors if the appropriate and cultivated culture-tailed strategies are used. Physical inactivity is a modifiable risk factor for several chronic conditions and a leading cause of premature mortality. An increasing proportion of adults worldwide do not engage in a level of physical activity sufficient to prevent or alleviate these adverse effects [13]. It is time, therefore, that we had nurse-led primary healthcare with an emphasis on health promoting strategies for disadvantaged people living in high behaviour-related cancer areas.

A few limitations must be considered when interpreting our findings. First, the cross-sectional nature of the data poses a limitation and prevents the inference of causal relationships. Second, self-reporting often underestimates true alcohol intake; and because there were more women participants in this study, the prevalence of ABC users might have been an underestimate. Third, several unaccounted factors (i.e., exposure to media messages about tobacco or alcohol or betel-nut, price or cost of ABC, psychiatric disorders, and use of other substances) could have affected the residents’ ABC behavior.

## Conclusion

This is the preliminary outcome of a nursing faculty’s longitudinal cohort study that cooperated with a multidisciplinary research team in an area of high prevalence of behavior-related cancer. It has identified that a high percentage of community adults living around the western coastal region regularly consumed ABC. Betel-nut and cigarette users were usually male, middle aged, with a poor attitude to health promoting behavior. Betel nut chewers are likely to smoke and drink, and usually do not take part in regular physical activity and have poor oral hygiene. Further research is required to understand the reasons why the subjects consume ABC, and explore ways to prevent initiation and enhance cessation of ABC habits in this population.

## Competing interests

The authors hereby declare that there were no competing interests.

## Authors’ contributions

SEG: Conceptualization of the study, study design, data analysis, discussion and editing of the final draft for publication. TJH: Conceptualization of the study and data collection. JCH: Data collection. MSL: Data collection. RMH: Editing of the final draft for publication. CHC: Data analysis. MYC: Conceptualization of the study, study design, proposal writing, data analysis, discussion and editing of the final draft for publication. All authors read and approved the final manuscript.

## Pre-publication history

The pre-publication history for this paper can be accessed here:

http://www.biomedcentral.com/1471-2458/13/257/prepub
